# Case of Pseudo-Membranous Laryngo-Bronchitis, or Croup, in an Adult

**Published:** 1852-08

**Authors:** 


*Case of Pseudo-Membranous Laryngo-Bronchitis, or Croup, in an Adult.* By M. Bouillaud. *Clinical Remarks on Laryngo-Bronchial Diphtheritis, or Croup, in adults.* By Dr. Bonnet.—The subject of M. Bouillaud's case was a pregnant female, aged 33, of impaired constitution, who was seized, on the 4th February, with symptoms of inflammation of the larynx and bronchi. On the 10th, she miscarried, and soon after died exhausted. A *post-mortem* examination disclosed signs of inflammation in the air-passages, in which were found also a quantity of false membrane. During her illness she had coughed up some casts of the trachea and bronchi; and M. Bouillaud thinks that death was rather due to the exhaustion produced by the miscarriage.

Dr. Bonnet relates two cases, also occurring in pregnant females. One of them died; the other recovered, and subsequently gave birth to a living infant. He does not think that diphtheritic exudation is always connected with inflammation. The only rational treatment is by emetics, which act, first, by causing mechanical expulsion; and secondly, by augmenting the secretion of the air-tubes, so as to favor the softening and removal of the false membrane. Their production of diaphoresis is also valuable. In cases of imminent danger, Dr. Bonnet would perform tracheotomy.—*London Journal of Medicine, June,* 1852.

